# Screening for precancerous anal lesions with P16/Ki67 immunostaining in HIV-infected MSM

**DOI:** 10.1371/journal.pone.0188851

**Published:** 2017-11-30

**Authors:** Sergio Serrano-Villar, Beatriz Hernández-Novoa, Amparo de Benito, Jorge del Romero, Antonio Ocampo, José Ramón Blanco, Mar Masiá, Elena Sendagorta, Gonzalo Sanz, Santiago Moreno, José A. Pérez-Molina

**Affiliations:** 1 Department of Infectious Diseases, Facultad de Medicina, Hospital Unversitario Ramón y Cajal, Universidad de Alcalá, IRYCIS, Madrid, Spain; 2 Department of Histopathology, Hospital Universitario Ramón y Cajal, Madrid, Spain; 3 Centro Sandoval, Madrid, Spain; 4 HIV Unit, Hospital Universitario de Vigo, Vigo, Spain; 5 Hospital San Pedro Centro de Investigación Biomédica, Logroño, Spain; 6 Infectious Diseases Unit, Hospital Universitario de Elche, Elche, Spain; 7 Department of Dermatology, Hospital Universitario La Paz, Madrid, Spain; 8 Department of General Surgery, Hospital Universitario Clínico San Carlos, Madrid, Spain; Rudjer Boskovic Institute, CROATIA

## Abstract

**Background:**

Screening of anal cancer in HIV-infected MSM with anal cytology results in high rates of false positive results and elevated burden of high-resolution anoscopies. High-risk HPV up-regulates p16 and Ki67 expression in epithelial cells. We assessed the usefulness of P16/Ki-67 immunostaining cytology for the diagnosis of precancerous anal lesions.

**Methodology:**

Cross-sectional multicenter study. Concomitant anal liquid cytology with p16/Ki-67 immunostaining and HRA with biopsy of acetowhite lugol-negative lesions was performed in HIV-infected MSM. We compared the diagnostic performance of an abnormal anal cytology and p16/Ki-67 immunostaining relative to HRA-guided biopsy by logistic regression and comparison of ROC areas.

**Results:**

We included 328 HIV-infected MSM. HSIL was histologically diagnosed in 72 subjects (25.1%), and 2 (0.6%) were diagnosed with anal cancer. An abnormal cytology showed a sensitivity of 95.6% and a specificity of 58.8% for the diagnosis of biopsy-proven HSIL. P16/Ki67 positivity was associated with the presence of biopsy-proven HSIL (P trend = 0.004) but with low sensitivity (41.2%) and specificity (71%). The combination of standard cytology with P16/Ki67 immunostaining did not increment the predictive value of standard cytology alone (AUC 0.685 vs. 0.673, respectively, P = 0.688).

**Conclusion:**

In HIV-infected MSM P16/Ki67 immunostaining does not improve the diagnostic accuracy of anal cytology, which shows a high sensitivity yet poor specificity. Other approaches aimed at improving the diagnostic accuracy of current techniques for the diagnostic of precancerous HSIL are warranted.

## Introduction

Although uncommon in the general population, anal cancer has emerged as a leading neoplasia among people living with HIV [1,2]. This has been especially documented in MSM, in whom incidence rates between 15 to roughly 100 times the rate observed in the general population have been reported [3]

Wherever resources are available, the current screening strategy is based on the detection of high-grade squamous intraepithelial lesions (HSIL), a cancer precursor, using anal cytology [4]. While this approach is sensitive, the specificity is poor [5,6], leading to an excess in the number of invasive procedures, including unnecessary high resolution anoscopies (HRA) and anal biopsies, patient discomfort and increased costs. Therefore, new diagnostic procedures are warranted to overcome the limitations of the anal cytology-based screening in susceptible populations.

Intense Human papillomavirus (HPV) biomarker research is undergoing to improve the accuracy of cervical and anal cytology-based screening programs. In epithelial cells, high-risk HPV up-regulates p16 expression via the viral oncoprotein E7 [7] and induces Ki67 expression, reflecting increased epithelial proliferation. Co-expression of both proteins indicates cell cycle de-regulation [8], has been associated to progression of cervical HPV to HSIL [9], and has been shown to reduce the number of unnecessary colposcopies in women [10]. Hence, this biomarker might also help to improve anal cancer screening. We assessed the usefulness of P16/Ki-67 dual staining cytology for the diagnosis of precancerous anal lesions in HIV-infected MSM.

## Materials and methods

### Study population

We conducted a prospective multi-center cohort study in 5 HIV clinics (Hospital Ramón Cajal, Hospital de Vigo, Hospital San Pedro, Hospital de Elche, Hospital La Paz, and Hospital Clínico San Carlos) and an STI Clinic (Centro Sandoval) in Spain from March 2013 to September 2016. We included HIV-infected MSM who were >18 years of age and who were not diagnosed with anal cancer prior to enrolment. This study conformed to the principles of the Declaration of Helsinki and Good Clinical Practice Guidelines and was approved by the Independent Ethics Committee of the coordinating center (Hospital Ramón y Cajal). All the participants gave informed consent before initiation of the study procedures.

### Anal cytology

A Dacron^®^ cytobrush moistened with tap water was used to sample the anal canal, and was rinsed in a vial containing 20 ml of PreservCyt (Hologic, Inc., Marlborough, MA, United States) fixative medium. The Bethesda System (TBS 2001) criteria were used for cytology reporting [11]. Cytological results were classified as unsatisfactory, negative, atypical squamous cells of undetermined significance (ASCUS), low-grade squamous intraepithelial lesion (LSIL), or HSIL. The same ThinPrep slide was used for p16/Ki-67 immunostaining after the cytologic evaluation with the CINtec PLUS Kit (Roche MTM Laboratories). A case was considered positive if one or more squamous epithelial cell(s) stained both with a brown cytoplasmic stain (p16) and a red nuclear stain (Ki-67) irrespective of any morphologic abnormalities. The number of cells that were positively stained with both markers was recorded. All samples were stained with p16/Ki-67 regardless of slide cellularity.

### HRA and histology

Concomitant HRA with biopsy of acetowhite lugol-negative lesions or suspicious of HSIL was offered for all participants. HRA was performed using the standard procedure, including topical application of 3% acetic acid and lugol solution in the anal canal. Anal biopsies were taken from suspicious areas revealed by HRA as acetowhite lugol-negative lesions with baby-Tischler forceps. The histologic results were been classified according to the Lower Anogenital Squamous Terminology (LAST) project recommendations [12]. Results were reported as negative, LSIL, HSIL, or squamous cell carcinoma. When multiple biopsies were obtained, the most severe result was used as the histo- logical diagnosis in the analysis. Treatment with infrared coagulation was offered to patients in whom the biopsy revealed high-degree intraepithelial lesions (HSIL). Biopsies were not obtained in participants who were evaluated as having no visual abnormalities suggestive of SIL on HRA examination, who were classified as being negative for SILs.

### Statistical methods

The outcome of interest was the composite variable of biopsy-proven HSIL, for which a negative outcome was considered whenever the HRA examination was normal and no biopsies were taken or a biopsy showed less than HSIL. We used variance-weighted least-squares regression to calculate the linear trends of the association between p16/Ki-67 positivity and the presence of histologic abnormalities. To compare the diagnostic performance of an abnormal anal cytology (atypical squamous cells, LSIL or HSIL) and p16/Ki-67 positivity relative to HRA-guided biopsy, we first calculated the sensitivity, specificity, positive predictive value and negative predictive value of each technique independently and the combined values of an abnormal cytology in combination with p16/Ki-67 positivity. We further examined the predictive ability of the standard cytology *vs*. p16/Ki-67 immunostaining by fitting a multivariate logistic regression model. In model 1, both variables were considered as predictors and the presence of biopsy-proven HSIL was the composite outcome as defined above. We performed sensitivity analyses to test whether the predictive value of p16/Ki-67 immunostaining changed when using the threshold of >4 and >10 positive cells or when excluding the samples with insufficient slide cellularity. In model 2, we repeated the previous steps but we only considered included as a predictor the abnormal cytology variable. Finally, we tested whether p16/Ki-67 immunostaining incremented the predictive value of the liquid-based cytology by using a test of equality of the ROC areas of both Models 1 and 2.

## Results

### Description of the study population

We included 328 HIV-infected MSM. Mean age was 39±10 years. Median nadir of CD4^+^ T-cell count was 367 cells/mm^3^ (258, 510), median CD4^+^ T-cell count 602 cells/mm^3^ (470, 798) and median CD4/CD8 ratio 0,47 (0.04, 0.80). Most patients were on ART (74.6%) and 57% had plasma HIV RNA levels below 20 copies/mL. Nearly half of the participants were smokers. The median number of lifetime sexual couples was 100 (20, 300), 47% reported condomless anal intercourses within the previous 3 months and 24% reported condomless sex in association with chemsex use (Table 1).

**Table 1:** General characteristics of the study population.

	N = 328
**Age, years [mean(SD)]**	39 (10)
**Male [N (%)]**	328 (100%)
**Tobacco use [N (%)]**	161 (49%)
**Nadir CD4, cells/uL (p50, IQR)**	367 (258, 510)
**CD4, cells/uL (p50, IQR)**	602 (470, 898)
**CD4/CD8 ratio (p50, IQR)**	0.47 (0.04, 0.80)
**On ART (N, %)**	245 (74.6%)
**HIV RNA <20 copies/mL**	186 (57%)
***Sexual practices***	
**No. of lifetime sexual couples (p50, IQR)**	100 (20, 300)
**No. of sexual couples in the past 12 months (p50, IQR)**	3 (1, 15)
**Last condomless anal intercourse in the past 3 months [N (%)]**	70 (30%)
**Condomless sex with concomitant chemsex use [N (%)]**	78 (24%)

### Anal cytologies, histology and P16/Ki67 immunostaining

All subjects received an anal cytology. Overall, 111 (33.8%) subjects had negative findings, 24 (7.3%) had ASCUS, 119 (36.3%) had LSIL, and 47 (14.3%) had HSIL. From the 328 subjects undergoing HRA, 41 (12.5%) were deemed normal and did not have a biopsy in the study, 147 (51.2%) were negative, 61 (21.3%) proved LSIL, 72 (25.1%) HSIL and 2 (0.7%) showed carcinoma.

A total of 237 patients were fully evaluable, in terms of satisfactory cytology, P16/Ki67 immunostaining and successful HRA with evaluable biopsies. Table 2 summarizes the positivity of p16/Ki-67 immunostaining in the histologic anal disease categories. A total of 76 from 237 (32.1%) men were positive for p16/Ki-67 immunostaining. There was a significant trend of increasing percentage of men testing positive for p16/Ki67 immunostaining with increasing histologic severity of disease, from 23% of men without dysplasia to 41% for men with HSIL (P trend = 0.004).

**Table 2:** Frequencies of P16/Ki67 positive immunostaining according to histologic results.

	Histology				
Cytology	*Unsatisfactory*	*Negative*	*LSIL*	*HSIL*	*Total*
***P16/Ki67 (-)***	6 (85.7%)	77 (77%)	38 (61.3%)	40 (58.8%)	161 (67.9%)
***P16/Ki67 (+)***	1 (14.3%)	23(23%)	24 (38.7%)	28 (41.2%)	76 (32.1%)
***Total***	7 (100%)	100 (100%)	62 (100%)	68 (100%)	237 (100%)

P trend = 0.004.

P16/Ki67 immunostaining was not obtained in 12/80 biopsy-proven HSIL.

### Predictive value of liquid-based cytology with and without P16/Ki67 immunostaining for the diagnosis of biopsy-proven HSIL

Using atypical squamous cells or worse as the threshold to consider an abnormal cytology, we found that the sensitivity and specificity of the standard cytology for the diagnosis of biopsy-proven HSIL was 96% and 42%, respectively, while for P17/Ki67 immunostaining the values were 42% and 61% (Table 3).

**Table 3:** Predictive values for the diagnosis of biopsy-proven HSIL.

	Abnormal cytology	PI16/Ki67 positivity	PI16/Ki67 positivity + abnormal cytology
**Sensitivity (95% CI)**	95.6% (91.2–99.9)	41.2% (29.2–53.1)	42.6% (29.9–55.4)
**Specificity (95% CI)**	58.8% (52.2–65.4)	71.0% (73.9–78.1)	61.1% (50.8–71.4)
**Positive predictive value (95% CI)**	39.8% (33.2–46.4)	37.3% (26.1–48.5)	42.6% (29.8–55.4)
**Negative predictive value (95% CI)**	95.8% (91.6–99.9)	25.8% (18.8-32-8)	38.9% (28.6–49.2)

Abnormal cytology: ASCUS, LSIL or HSIL.

To evaluate whether P17/Ki67 immunostaining may, however, add predictive value when combined with standard cytology, we fitted a predictive logistic regression model (Table 4). While an abnormal cytology was highly predictive of HSIL (OR 16.3, 95% CI3.6–77.3), a positive P16/Ki67 immunostaining was not associated to HSIL (OR 1.18; 95% CI 0.6–2.5).

**Table 4:** Logistic regression analysis. Outcome: Biopsy-proven HSIL.

*Model 1*				
		Odds ratio	95% CI	P value
**Abnormal cytology**	All samples	10.3	3.0–35.2	<0.001
	*Sensitivity analyses*			
	P16/Ki67 cutoff >4 cells	9.8	2.8–33.9	<0.001
	P16/Ki67 cutoff >10 cells	12.9	2.9–57.6	0.001
	Excluding insufficient cellularity	10.3	3.0–35.2	<0.001
**P16/Ki67 positivity**	All samples	1.2	0.6–2.5	0.648
	*Sensitivity analyses*			
	P16/Ki67 cutoff >4 cells	1.2	0.7–2.6	0.661
	P16/Ki67 cutoff >10 cells	0.7	0.2–2.6	0.544
	Excluding insufficient cellularity	1.2	0.6–2.5	0.648
*Model 2*				
**Abnormal cytology**		10.7	3.2–36.3	<0.001

The combination of the standard cytology with P16/Ki67 immunostaining did not increment the predictive value of standard cytology alone (AUC 0.685 vs. 0.673, respectively, P = 0.688).

**Fig 1:**
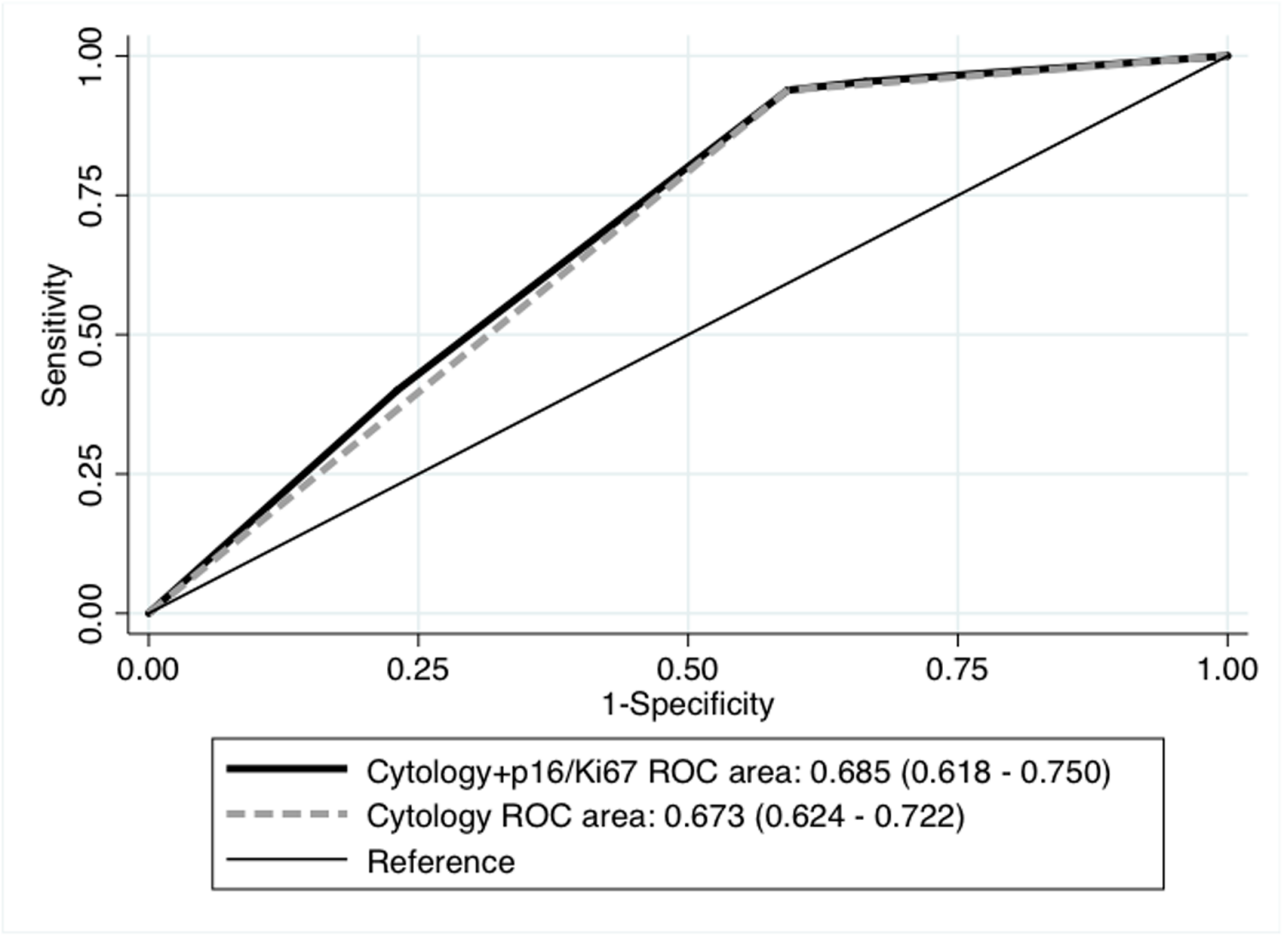
ROC area for diagnosis of biopsy proven-HSIL of the combination of the standard cytology with P16/Ki67 immunostaining vs. standard cytology alone.

Further, the combination of the standard cytology with P16/Ki67 immunostaining did not increment the predictive value of standard cytology alone (AUC 0.685 *vs*. 0.673, respectively, P = 0.688) (Fig 1).

These results remained unchanged in the sensitivity analyses for a positive P16/Ki67 test using a threshold of >4 positive cells, >20 positive cells or when excluding the samples with insufficient slide cellularity.

## Discussion

Here, we show in a cohort of HIV-infected MSM that P16/Ki67 immunostaining does not improve the diagnostic performance of standard liquid-based cytology for the diagnosis of biopsy-proven HSIL. In contrast, ongoing research on this cellular marker has yield promising results in the field of cervical HSIL detection. This biomarker might enhance the sensitivity [13,14] and specificity [15] of cervical cytology, and p16/Ki67 testing could reduce referral to colposcopy to almost half while detecting the most severe cases of HSIL[10]. In a large trial of 27,456 women in Europe, it was also associated to superior sensitivity but comparable specificity to standard cytology [16].

Compared to cervical HPV disease, data on usefulness of P16/Ki67 staining for the screening of HSIL in HIV-infected MSM remain very scarce. In a cohort of 363 HIV-infected MSM in the US, Wentzensen *et al*. found that the sensitivity and specificity of p16/Ki67 immunostaining for the diagnosis of histologic AIN3 were 93% and 38%, respectively [17]. The authors proposed using more than one dual-stain positive cells as the cut-off point, which yielded increased specificity of 55% while maintaining a sensitivity of 89%. In addition, in a French cohort study of 120 HIV-infected patients, p16/Ki67 positivity had a sensitivity of 64% and a specificity of 90% in detection of LSIL and HSIL indicated by anal cytology [18]. More recently, the Study of Prevention of Anal Cancer (SPANC) in Australia has found a sensitivity of 90% and a specificity of 51% for the diagnosis of HSIL [19]. Of note, in both studies a composite disease endpoint defined as the presence of HSIL-AIN3 on either cytology or histology was used, likely affecting the observed prevalence of HSIL and hence the predictive performance of P16/Ki67 immunostaining. Since we have found in our clinical experience that rectal sexually transmitted infections often result in cytological abnormalities that normalize following treatment, we decided to be conservative in our definition of HSIL. We found, however, a prevalence of biopsy-proven HSIL (29%) within the range of the other two studies (22% and 39%)[10,19]. Differences in the sampling techniques (anal swab *vs*. cytobrush), the performance of the HRA providers and the definition of the assessed outcomes might explain the differences in the predictive values reported across the different studies.

In our work, standard cytology showed a good sensitivity, yet the specificity was poor, and P16/Ki67 immunostaining failed to improve the detection of HSIL. For reasons poorly understood, the specificity of most candidate biomarkers is lower in HIV-infected than in uninfected individuals [19], and in contrast to what has been reported in the cervix, our data argue against the usefulness of P16/Ki67 immunostaining in anal cancer screening.

In conclusion, the low specificity of p16/Ki67 immunostaining found in HIV-infected patients indicates that this technique might hardly prove to be cost-efficient for anal cancer screening in HIV-infected subjects. Novel and accurate biomarkers are needed for the detection of precancerous HSIL. Identifying high-risk patients would reduce unnecessary HRA, diagnostic work-ups and medical visits, as well as, decrease patients’ concern during the follow-up.
